# On the Permeation of Polychlorinated Dibenzodioxins and Dibenzofurans through Lipid Membranes: Classical MD and Hybrid QM/MM-EDA Analysis

**DOI:** 10.3390/membranes13010028

**Published:** 2022-12-25

**Authors:** Raúl Alvarado, Gustavo Cárdenas, Juan J. Nogueira, Nicolás Ramos-Berdullas, Marcos Mandado

**Affiliations:** 1 Department of Physical Chemistry, University of Vigo, Lagoas-Marcosende s/n, 36310 Vigo, Spain; 2 Department of Chemistry, Universidad Autónoma de Madrid, Calle Francisco Tomás y Valiente, 7, 28049 Madrid, Spain; 3 IADCHEM, Institute for Advanced Research in Chemistry, Universidad Autónoma de Madrid, Calle Francisco Tomás y Valiente, 7, 28049 Madrid, Spain

**Keywords:** dioxins, lipid membranes, molecular dynamics, QM/MM, EDA

## Abstract

The permeation of dioxin-like pollutants, namely, chlorinated dibenzodioxins and dibenzofurans, through lipid membranes has been simulated using classic molecular dynamics (CMD) combined with the umbrella sampling approach. The most toxic forms of chlorinated dibenzodioxin and dibenzofuran, 2,3,7,8-tetrachloro-p-dibenzodioxin (TCDD) and 2,3,7,8-tetrachlorodibenzofuran (TCDF), and a dioleyl-phosphatidylcholine (DOPC) lipid membrane of 50 Å wide have been chosen for our study. The free energy profile shows the penetration process is largely favoured thermodynamically (ΔG ≈ −12 kcal/mol), with a progressively decrease of the free energy until reaching the energy minima at distances of 8 Å and 9.5 Å from the centre of the membrane for, respectively, TCDD and TCDF. At the centre of the membrane, both molecules display subtle local maxima with free energy differences of 0.5 and 1 kcal/mol with respect to the energy minima for TCDD and TCDF, respectively. Furthermore, the intermolecular interactions between the molecules and the lipid membrane have been characterized at the minima and the local maxima using hybrid quantum mechanics/molecular mechanics energy decomposition analysis (QM/MM-EDA). Total interaction energies of −17.5 and −16.5 kcal/mol have been found at the energy minima for TCDD and TCDF, respectively. In both cases, the dispersion forces govern the molecule-membrane interactions, no significant changes have been found at the local maxima, in agreement with the classical free energy profile. The small differences found in the results obtained for TCDD and TCDF point out that the adsorption and diffusion processes through the cell membrane are not related to the different toxicity shown by these pollutants.

## 1. Introduction

Chlorinated dibenzo-p-dioxins and dibenzofurans (CDDs and CDFs), together with certain polychlorinated biphenyls (PCBs), constitute a family of highly toxic compounds, known as dioxins, whose carcinogenic and mutagenic effects in living organisms are widely known [1,2,3,4,5]. They are considered persistent organic pollutants (POPs) and have an extensive list of production sources, including incineration of waste, production of iron and steel, chlorine bleaching, sewage sludge, or even natural processes such as forest fires, among others [6,7,8]. Due to their extensive production and high persistence in nature, CDDs and CDFs pose a serious risk to environmental health. Among the most toxic CDDs and CDFs, the tetrachlorinated forms of 2,3,7,8-tetrachloro-p-dibenzodioxin (TCDD) and 2,3,7,8-tetrachlorodibenzofuran (TCDF) may be remarked [9,10]. Their toxicity has been extensively investigated and has been mainly related to the affinity of dioxins by the AhR (aryl hydrocarbon receptor) cytosolic protein [11,12,13]. However, their toxicity is also connected to their high bioaccumulation within the fat tissues of living organisms [14,15], which causes the spread along the food chain by ingestion of animals or plants where they may be present in low concentrations [16].

Adsorption and diffusion of dioxins through the cell membrane are the first steps of its action mechanism within cells. The qualitative aspects of this mechanism have been extensively studied experimentally for TCDD, the most toxic dioxin, finding clear evidence that the binding of TCCD to AhR is the key stage that determines the toxicity to a greater extent, as mentioned above. AhR is a transcription factor that regulates the expression of different genes and is related to many disorders and diseases, such as major depressive disorder, cardiovascular disease, multiple sclerosis, rheumatoid arthritis, asthma, and allergic responses. Upon binding of dioxins to AhR, translocation into the nucleus and dimerization with ARNT (AhR nuclear translocator) may take place, provoking changes in gene transcription [11,12,13].

Although the qualitative aspects of the action mechanism of dioxins are relatively well-known, the mechanism itself has been scarcely investigated at the molecular level [17,18,19]. Particularly interesting are the adsorption and diffusion processes through the cell membrane and the role played by these membrane processes on the toxic levels detected for each CDD and CDF. Herein, computational simulations using classical methods provide very useful information about the thermodynamics and kinetics of the dioxin dehydration and diffusion steps along the membrane. Subsequent calculations at the hybrid quantum mechanics/molecular mechanics (QM/MM) level offer an accurate picture of the intra and intermolecular interactions that regulate the permeation of the membrane to different dioxins. Classical Molecular Dynamics (CMD) simulations of the penetration of TCDD through a lipid membrane have been already performed by Casalegno et al. [19]. They found the hydrophobic nature of TCDD favours its penetration within the structure of the lipid bilayer. A progressive decrease of the free energy profile from the water-lipid interface to the hydrophobic region was found, with a small increase of the free energy at the center of the membrane, leading to a subtle local free energy maximum. They also simulated the penetration of TCDD clusters of up to ten molecules, finding that the adsorption of the cluster molecules takes place sequentially but it is assisted by the intermolecular interactions among them. However, cluster disaggregation occurs just after adsorption so that TCDD migration across the membrane happens for each molecule independently of the rest.

As for CDFs, and more concretely, TCDF, no theoretical works have been published on its adsorption and membrane permeation or on its affinity for AhR to the best of our knowledge. TCDF presents some important structural and electronic differences with respect to TCDD, such as polarity and a partial reduction of local aromaticity of the phenyl rings, both induced by the replacement of the central dioxin-like ring by a furan-like ring. These structural and electronic differences may change the action mechanism with respect to TCDD, starting with the cell membrane crossing process. In fact, according to the toxic equivalency factors (TEFs), which were developed to account for the relative toxicity of dioxins, TCDD is significantly more toxic than TCDF [20].

Herein, a comparative study of the permeation of the TCDD and TCDF molecules through a lipid membrane model has been carried out using umbrella sampling combined with CMD. The aim of our work is twofold: on one hand, it intends to shed more light on the origin/nature of the intermolecular interactions governing the process, mainly at the free energy minima but also at other characteristic points, such as the local maxima, where the molecular environment changes due to the hydrophilic and hydrophobic regions of the lipid bilayer. On the other hand, a comparison with TCDF will provide valuable information about the role played by the membrane permeability on the relative toxicity of dibenzodioxins and dibenzofurans. In order to attain more insight into the process and understand the differences found between TCDD and TCDF, a hybrid QM/MM energy decomposition analysis (QM/MM-EDA) has been applied at specific regions along the permeation path (free energy minima and maxima) [21]. This is the first time the permeation of dioxins through biological membranes is studied at a quantum mechanical level. Thus, the results presented here serve also to check the ability of classical force fields to represent the intermolecular interactions of these molecules with the lipid bilayer.

## 2. Methodology

### 2.1. CMD Simulations

The membrane model employed for the CMD simulations consists of a lipid bilayer formed by two leaflet of 64 1,2-dioleyl-sn-glycero-phosphocholine (DOPC) molecules. Each layer is solvated by water molecules with a total thickness of 25 Å on each side. In order to reproduce the physiological concentration of KCl, chlorine anions and potassium cations were added until a concentration of 0.15 M. Casalegno et al. [19] employed a similar membrane model for their CMD simulations with a bilayer of 64 molecules each surrounded by water, but using as lipid unit 1,2-dipalmitoyl-sn-glycero-3-phosphocholine (DPPC), whose non-polar region differs slightly from that of DOPC. Phosphatidylcholine is one of the major phospholipid components of the plasma membrane and of the membrane of the endoplasmic reticulum, Golgi apparatus, mitochondria, endosomes, and lysosomes [22]. Due to the biological relevance of phosphatidylcholine lipids, DOPC and DPPC membranes are widely chosen as models in experimental and computational investigations. DOPC was chosen as a lipid unit by our group in previous studies of membrane permeability with very satisfactory results in comparison with the available experimental data [21,23]. The membrane was built with the help of the CHARMM-GUI website [24].

The reference CDD and CDF pollutants included in our study correspond to the more toxic forms, namely the TCDD and TCDF molecules. The molecules were initially placed at a distance of 32 Å along the normal direction from the centre of mass of the membrane with the help of the tleap module of AmberTools19 [25], giving rise to a system of 35,462 atoms. The Lipid17 force field, an updated version of the Lipid11 [26] and the Lipid14 [27] force fields, was employed to describe the lipid bilayer, whereas the TIP3P model was used to describe the water molecules [28]. K^+^ and Cl^−^ ions were represented by suitable Amber parameters [29]. The intramolecular and Lenard-Jones parameters of TCDD and TCDF were taken from the general Amber force field for organic molecules. To calculate the atomic charges, the geometry of the pollutants was initially optimized at the DFT level using the M062X [30] functional and the 6-311G(d,p) basis set, after which the Merz-Singh-Kollman scheme was applied at the same level of theory. CMD simulations were run with the pmemd CUDA implementation [31] of the Amber20 program [25].

Before the simulation of the pollutants’ membrane permeability, the equilibration of the structure and density of the solvated lipid bilayer was carried out. Initially, the system was minimized with the steepest descent method for 5000 steps, and the conjugate gradient method for another 5000 steps. Afterwards, it was progressively heated up to 303 K. In the first step, the system was heated to 100 K in the canonical (NVT) ensemble, using a Langevin thermostat with a collision frequency of 1 ps^−1^. In a second step, the system was heated from 100 K to the desired production temperature of 303 K at constant temperature and pressure (NPT ensemble), using again the Langevin thermostat and, in order to keep the pressure around 1 bar, the Berendsen barostat [32] with a pressure relaxation time of 1 ps. The positions of the lipid bilayer and the TCDD and TCDF molecules were restrained during the heating process by applying force constants of 10 kcal/(mol·Å^2^) and 5 kcal/(mol·Å^2^), respectively. Once the system temperature reached 303 K, two consecutive MD simulations of 4 ns each were carried out at constant temperature and pressure. During these two simulations, the restrictions to the position of the lipid molecules were reduced gradually by decreasing the force constant from 10 kcal/(mol·Å^2^) in the first simulation, to 5 kcal/(mol·Å^2^) in the second one. The force constant for the pollutants was kept at 5 kcal/(mol·Å^2^). As a final step in the equilibration process, a 100 ns production simulation was carried out in the NPT ensemble, keeping again the restrain force constant of the pollutants at 5 kcal/(mol·Å^2^) but removing the restrictions for the lipid positions.

**Figure 1:**
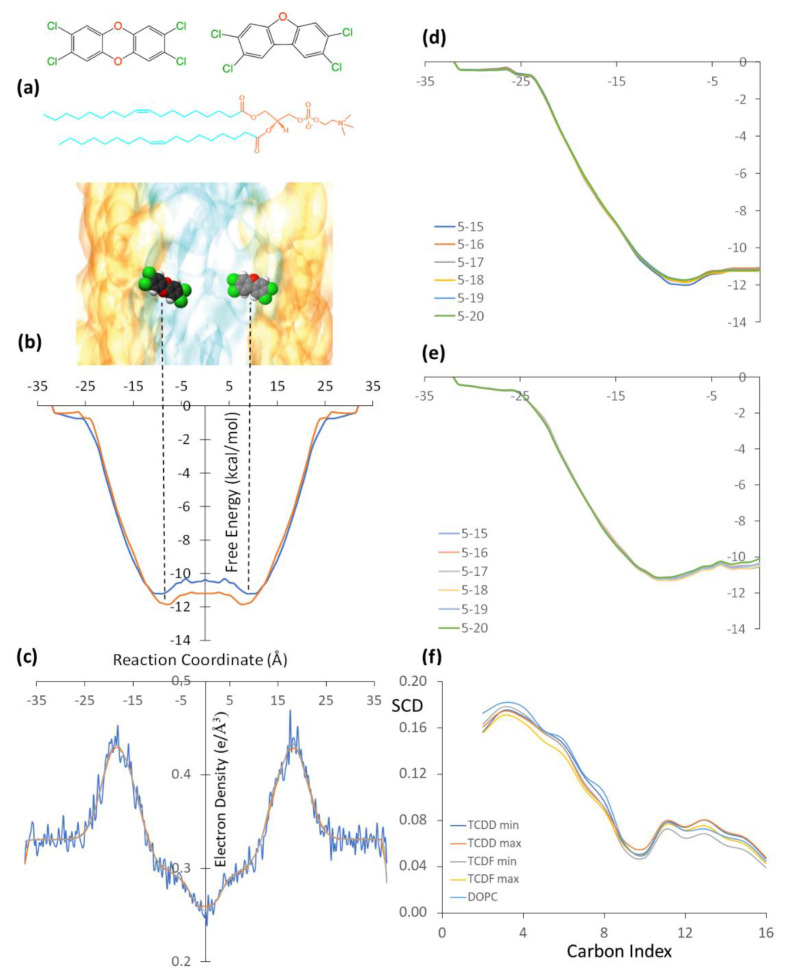
(**a**) Schematic representation of the TCDD (left) and TCDF (right) molecules embedded in a DOPC lipid bilayer. The polar and non-polar regions of the lipid bilayer are represented in orange and cyan, respectively. (**b**) Free energy profiles of the TCDD (red line) and TCDF (blue line) permeation processes through the lipid bilayer. The free energy minima are indicated by vertical dashed lines. (**c**) Electron density profile of the membrane along the reaction coordinate. (**d**) Free energy convergence analysis for TCDD using different simulation times per window, the time intervals are given in ns, and units of the free energies and reaction coordinates correspond to those displayed in the full free energy profile. (**e**) Free energy convergence analysis for TCDF using different simulation times per window. (**f**) Deuterium order parameter (SCD) of the acyl chains for the umbrella sampling windows corresponding to the energy minima and maxima for TCDD and TCDF and for a simulation of the membrane in absence of the TCDF and TCDD. Carbon atoms are numbered starting with the one closest to the polar head.

Once the system was equilibrated, the permeation processes of TCDD and TCDF through the lipid bilayer were simulated by means of the umbrella sampling technique. The distance between the centres of mass of the pollutant and membrane along the z-axis (perpendicular to the lipid bilayer) was defined as a reaction coordinate (see Figure 1), so that a value of 0 Å corresponds to the situation where the pollutant is located at centre of the membrane. A distance of 32.0 Å from the centre was taken as the initial value of the reaction coordinate. The whole reaction coordinate was divided into 65 windows of 0.5 Å width each. A CMD simulation of 20 ns was run within each window using the NPT ensemble. The initial geometry and velocities for a given window were taken from the last snapshot of the previous one. Additionally, a harmonic bias potential with a force constant of 2.5 kcal/(mol·Å^2^) was applied to the reaction coordinate. Thus, the full simulation of the pollutants’ penetration from the aqueous phase to the centre of the membrane took 1300 ns. The parameters employed for the simulation of each window were the same as those employed for the 100 ns production described above. The Weighted Histogram Analysis Method (WHAM) was employed to obtain the free-energy profiles [33,34]. During the full protocol, the particle-mesh Ewald method with a grid spacing of 1.0 Å was used to compute the electrostatic interactions, and a 10 Å cutoff for the nonbonded interactions was chosen. Moreover, the bonds involving hydrogen atoms were restrained by the SHAKE algorithm [35], and a time step of 2 fs was used. 

### 2.2. QM/MM-EDA Calculations

The interaction energy, $E_{\mathrm{int}}$, between two molecular systems A and B is defined as,

(1) $$E_{\mathrm{int}}=E_{\mathrm{AB}}-(E_{A}^{\mathrm{AB}}-E_{B}^{\mathrm{AB}})$$

where $E_{\mathrm{AB}}$ refers to the energy of the complex and $E_{A}^{\mathrm{AB}}$ and $E_{B}^{\mathrm{AB}}$ to the energies of the isolated systems calculated in the same geometry and with the same basis set as they have in the complex. 

The EDA scheme applied in this work for the study of non-covalent interactions is based on the partition of the complex energy, formulated in terms of the 1-electron density and density matrix and the exchange-correlation density (Equation (2)), into perturbed and unperturbed terms. Additionally, a second-order perturbation scheme is applied to decompose the polarization energy. The relevant theory related to this scheme can be found elsewhere, both for the QM-EDA [36,37] and the QM/MM-EDA [21,38] schemes. Herein, we will only mention that the total interaction energy of Equation (1) becomes partitioned into electrostatic (elec), Pauli repulsion (Pau), induction (ind), and dispersion (disp) energies.

(2) $$E_{\mathrm{int}}=E_{\mathrm{elec}}+E_{\mathrm{Pau}}^{}+E_{\mathrm{ind}}^{}+E_{\mathrm{disp}}^{}$$

In the study of the membrane-pollutant interactions, the system has been split into two subsystems, the pollutant molecule (A) and the DOPC membrane plus the water molecules and the K^+^ and Cl^−^ ions (B). As will be explained in the next section, the QM region includes the pollutant and a minimum of seven DOPC molecules, while the remaining atoms are included in the MM region and interact with the atoms of the QM layer by an electrostatic embedding approach. Thus, subsystem A is fully described at the QM level, whereas the complex and subsystem B are described at QM/MM level.

The Gaussian16 software package [39] has been employed for the QM/MM calculations. Electron densities in the QM region have been obtained using density functional theory (DFT), with the M062X functional and the 6-31G(d,p) basis set. This is an appropriate function to describe non-covalent interactions without the introduction of empirical dispersion corrections. To apply the QM/MM-EDA scheme explained above, three different electronic structure calculations are required; one for the complex (pollutant + solvated membrane), one for the solvated membrane, and another one for the pollutant molecule. The Gaussian input files for these calculations have been automatically generated with the MoBioTools package [40,41]. Both the pollutant and membrane input files include the full complex basis set in order to avoid the basis set superposition error in the QM region. As mentioned above, the QM/MM calculations were done using electrostatic embedding. Therefore, both the complex and membrane input files include the atomic charges of the MM region, which, together with the corresponding Cartesian coordinates, have been read from the Amber CMD simulations and written to the Gaussian input files using MoBioTools. The calculations of the interaction energy, its different components, and the analysis of the deformation densities have been performed with the EDA-NCI program [42].

## 3. Results and Discussion

As mentioned in the previous section, the permeation of pollutants through the membrane has been simulated using CMD combined with the umbrella sampling technique. The free energy profiles obtained by the WHAM from the simulations performed separately for TCDD and TCDF molecules are shown in Figure 1b, together with a scaled schematic representation of the DOPC bilayer (Figure 1a) and the electron density of the membrane along the reaction coordinate (Figure 1c). The simulation has been performed only for the left side of the path, from −35 Å to the centre of the membrane. Then, the free energy profile has been replicated on the right side of the plot in order to show the energy minima of the pollutants on different sides of the membrane. In addition, the right panel of Figure 1 also shows a convergence analysis (Figure 1d,e), where the free energy profile has been obtained by different simulation times per window. As can be seen, the profile is very well converged after running 20 ns in each window and, thus, it is not needed to extend the simulations.

The TCDD and TCDF energy profiles are very similar. The hydrophobic nature of the pollutants favours the permeation through the lipid bilayer with a progressive decrease of the free energy from the water-lipid interface to the hydrophobic region, which is identified by a maximum in the electron density plot, and from there to the hydrophobic region, where the electron density presents its lowest value. Moreover, a small increase of the free energy at the centre of the membrane is found in both cases, leading to subtle local free energy maxima. These profiles are in agreement with the CMD simulation performed by Casalegno et al. for the permeation of a TCDD molecule through a DPPC membrane. They found the free energy minimum around 10 Å from the centre of the bilayer, whereas our minimum is located around 8.0 Å. The energy well obtained by Casalegno et al. at the free energy minimum was around −10.3 kcal/mol, whereas the energy well obtained here is −11.8 kcal/mol. On the other hand, the free energy difference between the local maximum found at the centre of the membrane and the minimum was found to be around 1.7 kcal/mol in reference [19], whereas it is only 0.7 kcal/mol in this work. These small differences are likely related to the different membranes employed (DOPC vs DPPC), although the different computational details employed can also introduce some discrepancies. 

Looking now at the differences between TCDD and TCDF, the free energy minimum in the latter shows a small displacement with respect to the minimum of TCDD and it is located at 9.0 Å (and not at 8.0 Å) from the centre of the membrane. On the other hand, the free energy profile in the hydrophobic region of the lipid bilayer is slightly above the TCDD profile. The free energy minimum and the local maximum of TCDF are −11.2 and −10.4 kcal/mol, respectively, which are around 0.7 kcal/mol less negative than those obtained for TCDD. In other words, the permeation of TCDF is lowly less favourable than that of TCDD. This slight displacement to the hydrophilic region and slight destabilization inside the lipid bilayer with respect to TCDD could be explained by the small polar character of TCDF, with a calculated dipole moment of 0.59 D. The similarity between the two pollutants is also found in the behaviour of the acyl chains. In particular, the order of the lipid chains during the permeation processes was analysed in terms of the orientation of the C−H bonds with respect to the normal to the membrane by means of the so-called deuterium order parameter (SCD) of the acyl chains. The SCD parameter can go from 0 to 0.5, where a large value indicates a high degree of order of the acyl chains [26]. Figure 1f plots the SCD parameter for a simulation of the membrane in absence of the TCDF and TCDD and for the umbrella sampling windows located at the minimum and the maximum of the free energy profile of both pollutants. As can be seen, when both TCDF and TCDD diffuse through the membrane, the SCD parameter decreases for the atoms C1 to C9 with respect to the simulation where only the membrane is present. This indicates that the permeation of both molecules decreases the order of the acyl chain in the region close to the polar heads. The situation is slightly different for the atoms C10 to C16 of the acyl chain, i.e., for the atoms located in the nonpolar region of the membrane. In this case, the membrane order is smaller when TCDF is located at the minimum, while it is similar or slightly higher than the order of the isolated membrane for any other situation. This smaller degree of order for TCDF at the minimum is related to a favourable entropic contribution to the free energy. However, since the free energy is slightly more favourable for TCDD than for TCDF, one can conclude that the process is not governed by entropic factors but by enthalpic ones. In other words, the intermolecular interactions surpass the entropy contribution to the free energy and regulate the permeation mechanism. These intermolecular interactions are analysed in more detail in the following. 

In order to shed more light on the driving forces responsible for the TCDD and TCDF permeation and the slight differences encountered between them, an energy analysis of the intermolecular interactions between these molecules and the DOPC lipids has been performed around the free energy minima. This analysis could be performed by relying on the energy provided by the force field [21]. However, more accurate conclusions can be extracted if a QM/MM scheme is employed in the calculation and decomposition of the interaction energy, as was recently conducted for the permeation of cisplatin through a DOPC bilayer [21]. This accurate analysis requires the description of the interaction region using a QM level, but also the description of the environmental effects with a classical approach. As explained in Section 2, a hybrid QM/MM method including DFT for the QM region and an electrostatic embedding for the QM/MM interaction has been employed here to calculate the interaction energies. Subsequently, the electrostatic, Pauli, induction, and dispersion components of the interaction energy have been obtained with the QM/MM-EDA scheme mentioned in Section 2. The first step is to obtain the optimal size of the QM region for the QM/MM calculations. In our case, the optimal QM region must contain the pollutant and the minimum number of DOPC molecules (*N*_min_) necessary to obtain the pollutant-lipid total interaction energy and its different components converged with the region size. In order to obtain *N*_min_, one arbitrary snapshot has been selected around the free energy minimum of the CMD simulations of TCDD and TCDF permeation. Then, using these two geometries from these snapshots as input, QM/MM-EDA calculations have been performed for QM regions of an increasing number of DOPC molecules (*N*).

**Figure 2:**
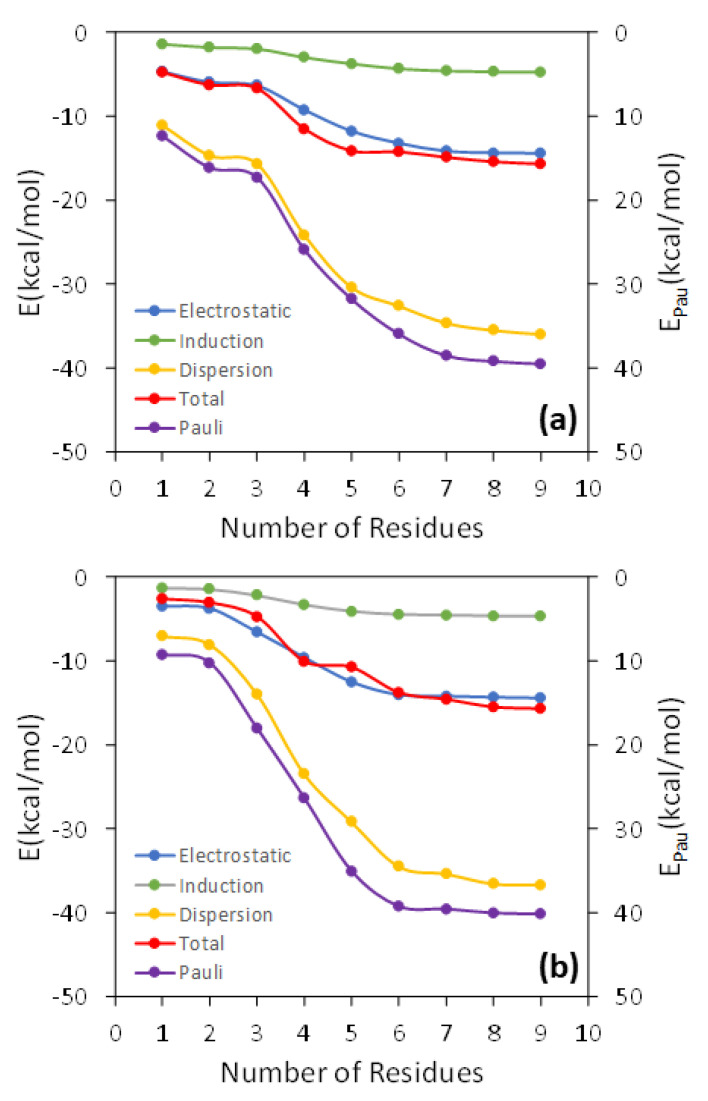
Analysis of the convergence of the interaction energy and its different components as the number of the DOPC molecules increases in the QM region for an arbitrary geometry around the free energy minima of the TCDD (**a**) and TCDF (**b**) permeation.

**Figure 3:**
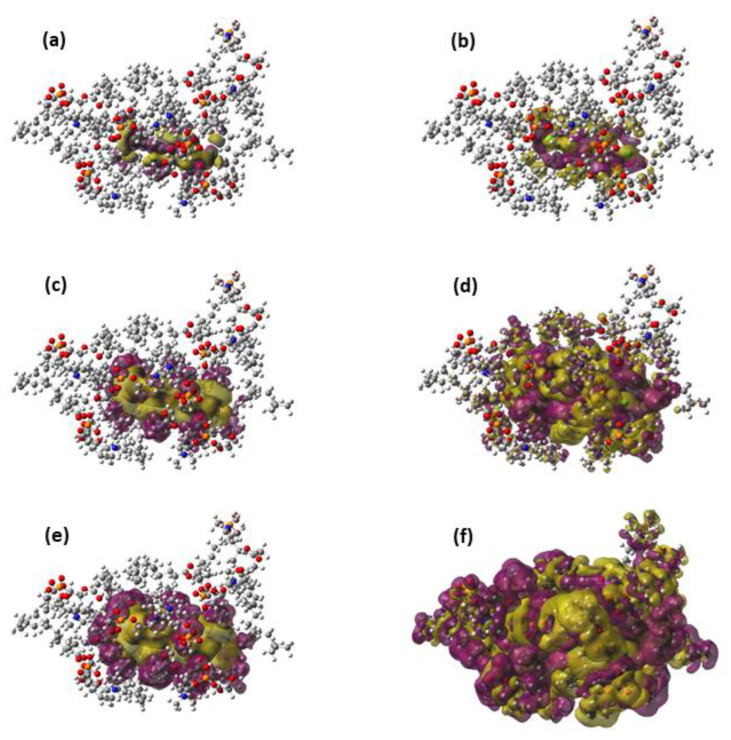
Representations of the Pauli deformation density (**a**,**c**,**e**) and the polarization density (**b**,**d**,**f**) for an arbitrary geometry around the free energy minimum of the TCDD permeation. The representation includes only the TCDD molecule and the seven DOPC molecules included in the QM region. The following density cut-offs have been used: 10^−4^ (**a**,**b**), 10^−5^ (**c**,**d**) and 10^−6^ (**e**,**f**).

In Figure 2, the total interaction energy as well as the electrostatic, Pauli, induction, and dispersion energies are represented against *N* for the interaction of TCDD and TCDF with the lipid bilayer. As can be observed, all the energy terms are converged within the interval of ±1 kcal/mol for *N* = 7, both in the case of TCDD and TCDF. It is remarkable that a slower convergence is not found for the electrostatic and induction energies. These terms correspond to the interactions with the largest range, and it could be expected that they need a larger number of DOPC molecules to converge than the shortest-range Pauli and dispersion energies. This occurs, for instance, in solvated anions, such as ammonium cation, formate anion, and the zwitterionic form of glycine [38]. However, this is not the case for the interaction of TCDD and TCDF with the lipid bilayer since the convergence is reached almost at the same time for all the energy terms. On the other hand, this result does not confront with the general assumption that Pauli energy is a short-range interaction. This character is clearly reflected in the magnitude of the Pauli deformation density, $\Delta \rho_{\mathrm{Pau}}$, which is represented using different density cut-offs and compared with the polarization density, $\Delta \rho_{\mathrm{Pol}}$, in Figure 3. As can be observed, $\Delta \rho_{\mathrm{Pol}}$ spreads over a larger region than $\Delta \rho_{\mathrm{Pau}}$ when the same cut-off is applied. $\Delta \rho_{\mathrm{Pol}}$ is involved in the calculation of induction and dispersion energies, whereas $\Delta \rho_{\mathrm{Pau}}$ only enters into the calculation of the repulsive part of the Pauli energy. The unexpected slow convergence of the Pauli and dispersion energies with the number of DOPC molecules may be related to the large contribution of these terms to the total interaction energy in comparison with the induction and electrostatic energies (see Figure 2). In fact, the dispersion energy is the strongest contribution among the attractive interactions for the two pollutants. This is in contrast to the solvated ions where the electrostatic energy is the dominant term in the interaction of the ion with the solvent [38].

Once *N*_min_ has been set to 7, QM/MM-EDA calculations have been carried out at the free energy minima and local maxima at the centre of the bilayer by sampling 100 geometries from each region from the previous CMD simulations. In a recent work on cisplatin permeation through the same lipid membrane, it was found that the mean values for the different interaction energy components were relatively well converged after considering 100 geometries. Taking into account the large computational cost of the QM/MM calculations performed here with a QM region size containing 988 atoms, we have selected 100 geometries equally spaced along the corresponding window for the QM/MM-EDA statistics.

The mean values obtained for the total interaction energy and its components at the energy minima and local maxima are collected in Table 1. As can be observed, the energies obtained at QM/MM level also predict TCDD is slightly more stable within the membrane than TCDF, in agreement with the classical free energy profile. In fact, the interaction energy difference is 1 kcal/mol, almost the same difference found for the free energies obtained classically in the dynamic study. Here, it should be recalled that the geometries used for the quantum mechanical study are those obtained classically as a QM/MM MD sampling is computationally unfeasible. Thus, possible differences arising from changes in the geometrical disposition of the pollutants within the lipid bilayer when going from a classical to the QM/MM MD sampling protocol are not accounted for by our calculations. However, it is remarkable the good correspondence between classical free energies and QM/MM interaction energies, both in absolute and relative terms. Differences in the total values can be mainly ascribed to entropic terms and thermal effects, which are not included in the interaction energies. Anyway, the entropic contribution to the pollutant permeation is likelydestabilizing, which is also reflected in the positive difference between classical free energies (−11.8 kcal/mol for TCDD and −11.2 kcal/mol for TCDF) and QM/MM interaction energies (−17.5 kcal/mol for TCDD and −16.5 kcal/mol for TCDF). As shown in Table 1, the stabilization of the pollutants within the membrane is clearly associated with the dispersion forces, which is the expected result taking into account the non-polar (TCDD) or slightly polar (TCDF) character of these molecules and their hydrophobic nature. However, although the electrostatic energy is less than half of the dispersion energy, it is not negligible. This may be due to the large-range character of this energy contribution and the relative proximity of the polar heads of the lipid molecules and the C-O polar bonds of the dioxin and dibenzofuran rings.

The EDA results obtained for the local maximum at the centre of the membrane reflect negligible differences with respect to the minimum for TCDF but more noticeable differences for TCDD. A possible explanation for this different behaviour may be found in the disposition of the polar and non-polar heads of the DOPCs in the lipid bilayer and the slightly different polarity and polarizability of TCDD and TCDF. Thus, the free energy local maximum is located at the centre of the bilayer, where the non-polar character is maximum, whereas the free energy minimum is somewhat displaced towards the polar region. In TCDF, the interaction energy difference with respect to the minimum is 1.5 kcal/mol in good agreement with the difference of 0.7 kcal/mol in the classical free energy. According to the results obtained for the different energy components, the slightly higher energy of the local maximum in TCDF arises from the Pauli and induction energies, which increases 2.0 and 0.4 kcal/mol, respectively, with respect to the minimum, which is not compensated by the decrease of the dispersion energy of 0.8 kcal/mol and the unchanged electrostatic contribution. On the contrary, an interaction energy increase of 3.5 kcal/mol with respect to the minimum is found for TCDD. Curiously, all the energy terms significantly decrease (in absolute value) in this case. This indicates the average distances between TCDD and the DOPC molecules increase slightly when TCDD moves towards the non-polar heads, whereas it keeps almost unaltered for TCDF. The percentages of change of the energies calculated at the local maxima with respect to the minima are shown in Table 1. From these percentages, it can be extracted that the largest relative changes with respect to the minimum in TCDD are found in the electrostatic and induction energies. The relative changes in the Pauli repulsion and dispersion are clearly smaller. Thus, the decreases in the Pauli energy when going from the minimum to the maximum (around 8 kcal/mol) issurpassed by the decrease of all the attractive contributions (mainly electrostatic and induction). On the other hand, the energy increase in the total interaction energy is significantly larger than that predicted by the CMD simulation. This could be caused by two reasons: (i) the force field performs badly when describing the interaction between TCDD and the membrane or (ii) there are important entropic effects that are not described by the QM/MM calculations.

**Figure 4:**
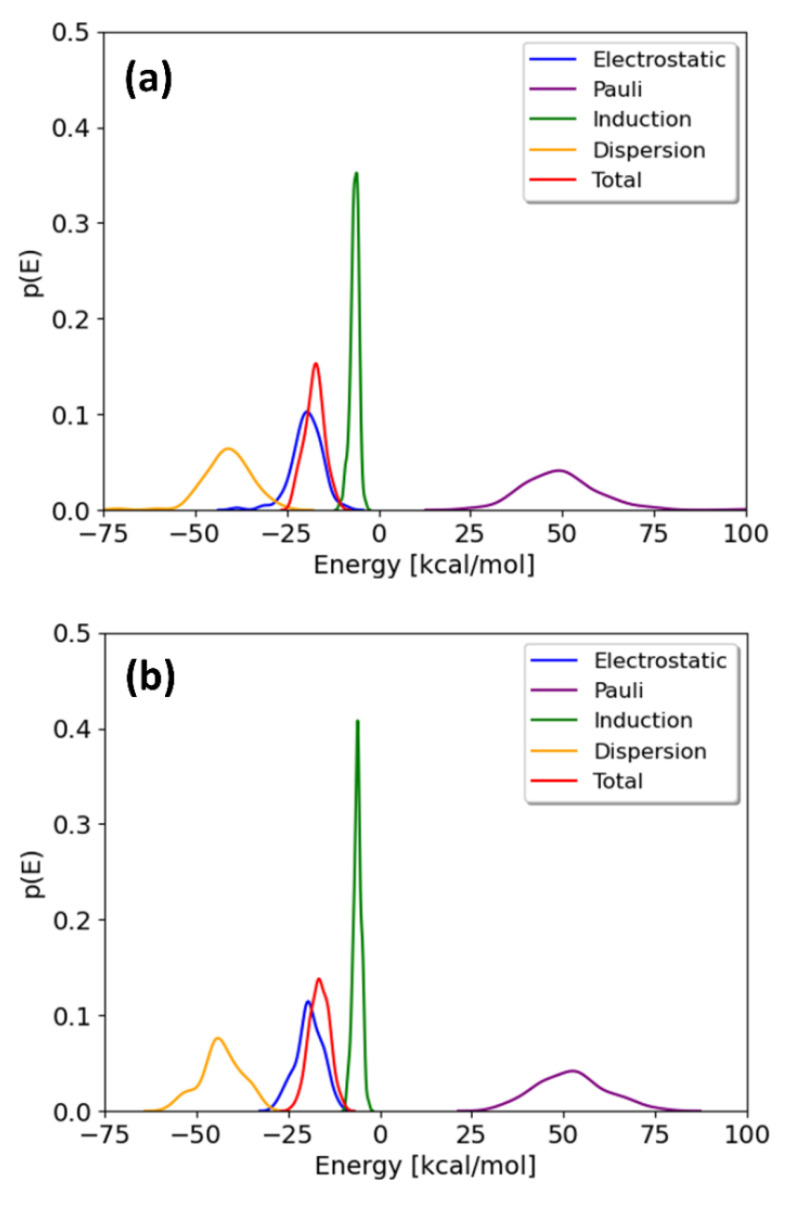
Distributions of the interaction energy and its different components on 100 geometries selected from the free energy minima of the TCDD (**a**) and TCDF (**b**) permeation.

The probability distributions of the total interaction energies and their energy components for TCDD and TCDF are depicted in Figure 4. They are only shown for the minima; the local maxima display basically the same profile. It can be observed that the distributions of the energy components present different broadness, whereas no significant differences are found between the pollutants. The broadness of the distributions seems to be associated with the magnitude of the average value, as previously found for the interaction of cisplatin with the same membrane [21]. The maxima of the distributions correspond to the mean values collected in Table 1. As can be seen, all the energy components but induction are significantly broad. This fact highlights the importance of performing a good sampling protocol when calculating the interaction energies for large systems, instead of relying on an arbitrary configuration of a local potential energy minimum. 

## 4. Conclusions

In this work, a comparative study of the permeation of TCDD and TCDF pollutants through a lipid membrane formed by DOPC molecules has been carried out using umbrella sampling CMD simulations and a QM/MM-EDA scheme. After the optimization of the QM region size, the QM/MM-EDA study has been performed on a statistical sample of geometries selected from the minima and local maxima regions of the free energy profile obtained from the CMD simulations. The QM/MM-EDA calculations performed in this work have revealed the nature of the intermolecular interactions between the pollutants and the lipid molecules that govern the permeation process. The comparison between dioxins of different toxicological activity has shed light on the role played by the membrane permeation step in their relative toxicity.

The CMD results show the permeation of TCDF is thermodynamically slightly favoured over TCDD (around 1 kcal/mol) and a slight displacement of the free energy minimum of TCDF to the hydrophilic region of the membrane with respect to TCDD. Both free energy profiles also display a local energy maximum at the centre of the membrane. The free energy differences with respect to the minimum are small in both cases (around 1 kcal/mol), a fact which reflects that the CMD simulations predict small changes in the lipid-pollutant interactions when crossing the hydrophobic region of the membrane or a compensation between the attractive and repulsive energy changes.

The optimal QM region size for the QM/MM-EDA study was found to be composed of the pollutant molecule and 7 DOPC molecules. The results obtained at QM/MM level are in good agreement with the CMD simulations. Thus, the total pollutant-membrane interaction energy at the minima indicates TCDD is more stable than TCDF by only 1 kcal/mol. However, the calculated QM/MM interaction energies are more negative than the free energies obtained classically, which could be associated with unfavourable entropic or thermal effects inside the membrane, which are taken into account in the classical simulations but not in the QM/MM interaction energy computations. The interaction energy calculated at the centre of the membrane for TCDF and TCDD displays differences with respect to the minimum only a bit larger than those obtained for the classical free energy, another proof of the good correspondence between classical and QM/MM calculations.

The analysis of the different interaction energy components reflects that the stabilization of the pollutants within the hydrophobic region of the membrane is mainly due to a strong dispersion interaction. In both pollutants, dispersion is more than twice the electrostatic energy, whereas the induction energy has a marginal contribution. On the other hand, the Pauli repulsion is also strong, largely compensating for the attractive energy terms. The higher interaction energy at the centre of the membrane has a different origin for TCDF and TCDD. In TCDF, a slight increase in the Pauli repulsion energy seems to be the main factor. On the contrary, all the terms decrease (in absolute value) with respect to the minimum in TCDD, being the significant reduction of electrostatic and induction energies, which are proportionally larger than Pauli and dispersion, the reason for the slight destabilization of TCDD at the membrane centre with respect to the minimum. 

As a general conclusion, the small differences encountered between the TCDD and TCDF permeation processes, both in the CMD simulations and the QM/MM-EDA calculations, points out that the adsorption and diffusion steps through the cell membrane do not explain the different toxicity measured for TCDD and TCDF.

## Figures and Tables

**Table 1:** Mean values of the interaction energy and its different components at the free energy minima and local maxima of the TCDD and TCDF permeation. The percentage of change with respect to the minimum of the energies at the local maximum is given in parentheses.

Min	Electrostatic	Pauli	Induction	Dispersion	Total
TCDD	−19.6	49.7	−6.5	−41.1	−17.5
TCDF	−19.3	52.1	−6.4	−43.0	−16.5
**Max**	**Electrostatic**	**Pauli**	**Induction**	**Dispersion**	**Total**
TCDD	−15.0 (23.5)	41.2 (17.1)	−4.7 (27.7)	−35.5 (13.6)	−14.0
TCDF	−19.3 (0.0)	54.1 (3.8)	−6.0 (6.3)	−43.8 (1.9)	−15.0
